# Occipital interhemispheric transtentorial approach to a pineoblastoma in a 4-year-old child

**DOI:** 10.3171/2021.4.FOCVID2122

**Published:** 2021-07-01

**Authors:** Alessia Imperato, Alessandra Marini, Pietro Spennato, Giuseppe Mirone, Giuseppe Cinalli

**Affiliations:** Department of Pediatric Neurosurgery, Santobono-Pausilipon Children’s Hospital, Naples, Italy

**Keywords:** pineoblastoma, child, occipital interhemispheric transtentorial approach, Herophilus-Galen line, neuroendoscopy

## Abstract

The authors present a pediatric case of a pineoblastoma treated with gross-total removal through an occipital interhemispheric transtentorial approach (OITA). The child presented with acute hydrocephalus that was treated by endoscopic third ventriculostomy (ETV) and tumor biopsy through a single burr hole. Histology revealed a pineoblastoma. Microsurgical total removal was performed 3 months after neoadjuvant chemotherapy. OITA was chosen on the basis of the tumor’s location below the Herophilus-Galen line of sight. In this video, the authors show the positioning, the operating devices, the approach, and the microsurgical dissection, indicating all the neurovascular structures encountered.

The video can be found here: https://stream.cadmore.media/r10.3171/2021.4.FOCVID2122.

## Transcript

In this video, we describe the occipital interhemispheric transtentorial approach to a pineoblastoma in a 4-year-old child. The patient is a 4-year-old baby girl with a history of headache and morning vomiting in the last month and drowsiness in the week before admission. Neurological exam at admission revealed speech delay and left sixth cranial nerve palsy. CT scan and craniospinal MRI showed triventricular hydrocephalus induced by a pineal mass without evident CSF seeding.

### 0:49 Radiology and Management Strategy

These are the images of MRI at presentation showing the midline pineal mass inducing obstructive triventricular hydrocephalus. It was decided to treat the hydrocephalus by endoscopic third ventriculostomy and biopsy the tumor with the single burr hole technique, allowing both procedures through the same approach.

### 1:06 Endoscopic Biopsy

After identification of the ideal trajectory for ETV joining the tuber cinereum and the center of Monro foramen, and identification of the ideal trajectory for tumor biopsy joining the tumor surface and the center of Monro foramen, we identify an entry point in the middle of the two trajectories allowing both procedures through a single burr hole. Endoscopic view shows the good visualization of both the floor of the third ventricle and of the tumor mass. The small white spots on the ependyma, although suspicious for seeding, were not biopsied because considered too small.

After standard third ventriculostomy, the endoscope is directed posteriorly, and the tumor is approached from behind the massa intermedia. Biopsy of the tumor mass can induce small bleeding that can be managed by delicate balloon inflation. At the end of the procedure, only minor contusions of massa intermedia and fornix are visible without clinical consequences. This is the immediate postoperative CT scan showing clean ventriculoscopy track with safety EVD that was removed 48 hours later. MRI performed 3 months after biopsy showed on T2, DWI, and dADC sequences absence of white matter damage in the right frontal lobe following the single burr hole procedure, to prove safety and feasibility of the technique in presence of anatomical conditions and with a good preoperative planning.

Histology revealed a pineoblastoma, CSF cytology was negative, and neoadjuvant chemotherapy with vincristine and carboplatine was administered that allowed significant reduction of the tumor volume. Neoadjuvant chemo is especially important in pineoblastomas that are highly hemorrhagic lesions, in reducing vascularity at radical surgery.

### 2:46 Selection of the Approach

Surgical removal of the residual tumor was decided, and two options of surgical approach were considered: the supracerebellar infratentorial approach and the occipital interhemispheric transtentorial approach.^1–10^ The SCIT approach can be uncomfortable in prone position, carries a risk of air embolism in sitting position. It is a fully infratentorial procedure with a deep narrow field, can be difficult in case of steep tentorium, sacrifice of bridging veins is necessary, and it is considered better for midline lesions.^6,8^ For the OITA approach, a preoperative lumbar drainage is preferable. It is performed in prone position, it’s a mainly supratentorial procedure offering a wide field after tentorium opening, it can be used independently on tentorium angle, venous sacrifice not always necessary, and offers a better lateral exposure.^4–6,8,9^

### 3:36 Herophilus-Galen Line

To select the approach, a sagittal midline cut is chosen. The lowest point of the vein of Galen is identified, then the highest point of the torcular Herophili is identified. The line joining these two points, the Herophilus-Galen line, identifies the highest possible line of sight of a microscope when performing an occipital interhemispheric transtentorial (OITA) approach. In this case, 100% of the tumor volume is located below this line and is therefore perfectly controlled through an OITA approach. This 3D reconstruction shows that the microscope view along the Herophilus-Galen line of sight offers perfect control of the upper part of the tumor, and adequate modification of the microscope working angle allows good control of the whole tumor volume. Torcular was very asymmetrical due to predominant right transverse sinus, so the tumor was approached through the left side.

### 4:31 Position, Skin Incision, and Craniotomy

Position is prone with head in 3-pin head frame turned 15° to the right. This 3D model shows the orientation of the surgical corridor from the surgeon’s point of view, the key structures of the torcular and the sagittal sinus, and the extension of the bone flap including the lambda and crossing the midline for the best possible control of the sagittal sinus. We prefer sigmoid incision, allowing easier median line dissection in the cervical region and larger occipitoparietal craniotomy. This are the bone exposure and the extent of the craniotomy flap with control of the torcular and sagittal sinus.

### 5:07 Interhemispheric Dissection

After opening of the lumbar drainage, interhemispheric dissection allows identification of the tentorium and quadrigeminal cistern. Tentorium is extensively coagulated with the bipolar, and then it is largely opened using the contact thulium laser.

### 5:22 Opening of Quadrigeminal Cistern

The arachnoid of the quadrigeminal cistern is exposed and opened using a Rhoton dissector or a sharp dissector. Larger opening of the arachnoid allows easy identification of the tumor tissue, and after completion of this step the dissection of the deep vein complex can start. After chemotherapy, the arachnoid can be very thick, so sharp dissection is necessary at least to open the outer layer of arachnoid that covers both the tumor and the deep vein complex, to avoid risk of traction or tearing of vessels.

### 5:54 Dissection of the Vein of Galen

The initial dissection allows unveiling of the vein of Galen and of the homolateral basal vein of Rosenthal, and the red dark mass of the tumor is well visible among them. After initial dissection and identification of the veins, tumor dissection can start, allowing identification of the superior vermian vein.

### 6:15 Tumor Debulking

The outer layer of the tumor is coagulated and opened sharply, and biopsy followed by internal debulking can be carried out with the ultrasonic surgical aspirator until visualization of the cavity of the third ventricle. Then we start to dissect the lateral extensions of the tumor, starting from the left pole that can be delicately removed by gentle fragmentation and aspiration with the ultrasonic device.

### 6:38 Dissection of Deep Veins

At this point, we improve our dissection of the deep vein complex. After chemotherapy, deep veins are embedded in a thick layer of arachnoidal tissue that can nevertheless be opened, allowing sharp dissection and identification of the vein of Galen, internal cerebral veins, basal vein of Rosenthal, and superior vermian vein. Here we are dissecting the left internal cerebral vein from the superior vermian vein. As you can see, the arachnoid is very thick, but sharp dissection allows identification of the dissection plan between the thick arachnoid layer and the wall of the veins, allowing dissection and identification of all the anatomical structures, avoiding excessive traction on the wall of the veins.

### 7:25 Sacrifice of the Superior Vermian Vein

After complete anatomical identification of the two Rosenthal, two internal cerebral veins, vein of Galen, and superior vermian vein, safer dissection of the upper pole of the tumor can be performed, after sacrifice of the superior vermian vein, that is the only vein of the complex that can be sacrificed with impunity if necessary. After this step, it is possible to have clear and larger access to the contralateral side, where the right component of the tumor can be finally dissected and removed.

### 7:55 Tumor Removal

Here the last adhesions of the last remnant of the tumor are disconnected and the tumor is removed, and this is the cavity of the third ventricle. The last remnants of the lower pole are easily identified on the tectal plate, carefully dissected, and removed at very high magnification.

### 8:11 Description of the Operative Field

The operative field is delimited by the splenium in the cranial part, the left internal cerebral vein, the contralateral basal vein of Rosenthal in the upper part of the operative field, and the homolateral basal vein of Rosenthal in the lower part. Vein of Galen is evident in the cranial part of the field at the attachment with the tentorium-falx junction. This is an overview of the surgical field at the end of surgery at lower magnification, showing the falx and the tentorium with the straight sinus running at the attachment. Occipital lobe surface is clean without contusions after patties removal.

### 8:46 Postoperative MRI

This is the postoperative MRI in axial cuts and sagittal cuts showing complete removal of the tumor. T2, DWI, and dADC sequences showed absence of cortical or white matter damage in the left occipital lobe due to retraction and absence of vascular problems related with the sacrifice of the superior vermian vein.

### 9:05 Clinical Outcome

The patient presented transient upward gaze palsy (Parinaud’s syndrome) without focal deficit. This is the patient dancing at her 6th day postoperatively. Asymptomatic left subdural hygroma was precautionarily treated by subduroperitoneal shunt before referring to high-dose chemotherapy with rescue peripheral blood stem cell transplantation. Craniospinal irradiation was administered with 54-Gy boost on tumor bed.

### 9:32 Conclusions

In conclusions, pineoblastomas are exceedingly rare entities. Initial endoscopic single burr hole technique is key for treatment of hydrocephalus and tissue sample. Neoadjuvant chemotherapy allows tumor shrinkage. The Herophilus-Galen line of sight is helpful in choosing surgical approach. Interhemispheric transtentorial approach allows complete removal of residual tumor without neurological deficits.

## Acknowledgments

We gratefully acknowledge Maria Rosaria Scala, MD, for help in 3D model reconstruction.

## Author Contributions

Primary surgeon: Imperato, Marini, Cinalli. Assistant surgeon: Imperato, Marini, Spennato. Editing and drafting the video and abstract: Imperato, Marini, Mirone, Cinalli. Critically revising the work: Imperato, Marini, Spennato, Cinalli. Reviewed submitted version of the work: Imperato, Marini, Cinalli. Approved the final version of the work on behalf of all authors: Imperato. Supervision: Cinalli.
